# Best practice advice on pre-hospital emergency anaesthesia & advanced airway management

**DOI:** 10.1186/s13049-018-0554-6

**Published:** 2019-01-21

**Authors:** Kate Crewdson, David Lockey, Wolfgang Voelckel, Peter Temesvari, Hans Morten Lossius, EHAC Medical Working Group

**Affiliations:** Norwegian Air Ambulance Foundation, Drøbak, Norway

## Abstract

**Background:**

Effective and timely airway management is a priority for sick and injured patients. The benefit and conduct of pre-hospital emergency anaesthesia (PHEA) and advanced airway management remains controversial but there are a proportion of critically ill and injured patients who require urgent advanced airway management prior to hospital arrival. This document provides current best practice advice for the provision of PHEA and advanced airway management.

**Method:**

This best practice advice was developed from EHAC Medical Working Group enforced by pre-hospital critical care experts. The group used a nominal group technique to establish the current best practice for the provision of PHEA and advanced airway management. The group met on three separate occasions to discuss and develop the guideline. All members of the working party were able to access and edit the guideline online.

**Results:**

This EHAC best practice advice covers all areas of PHEA and advanced airway management and provides up to date evidence of current best practice.

**Conclusion:**

PHEA and advanced airway management are complex interventions that should be delivered by appropriately trained personnel using a well-rehearsed approach and standardised equipment. Where advanced airway interventions cannot be delivered, careful attention should be given to applying basic airway interventions and ensuring their effectiveness at all times.

## Background

Airway management can be classified as basic (simple airway manoeuvres, naso- and oropharyngeal airways) or advanced (placement of a supraglottic airway device, cuffed tracheal tube or surgical airway with or without the provision of emergency anaesthesia). Basic airway interventions should be performed for every patient with airway compromise; emphasis must be placed on performing the intervention well, with repeated assessment of its effectiveness. In some patients, basic interventions will be insufficient to provide adequate oxygenation and ventilation. If appropriately trained personnel are available, advanced airway interventions should be performed, prior to transfer to hospital [1, 2]. The benefit and conduct of pre-hospital emergency anaesthesia (PHEA) is widely-debated topic and has been highlighted by an expert panel as an area to be prioritised in pre-hospital research [3]. Whilst the number of advanced interventions and success rates are increasing [4, 5], the practical aspects of advanced airway management in the pre-hospital setting are not internationally consistent. Many systems performing these interventions in the challenging pre-hospital environment are deficient in one or more key components that help to ensure high quality reproducible interventions. These include well-trained providers, standardised techniques, robust failed intubation drills, key data collection, and a clinical governance structure. The level of care delivered to patients in the pre-hospital setting must be the same as that achieved in hospital [6]. Physician-staffed helicopter emergency medical services (HEMS) have the potential to deliver advanced airway management at this level, but the essential elements must be in place.

Based on published scientific reports and guidelines [6, 7], the EHAC Medical Working Group (MWG) aims to provide ‘Best Practice Advice’ that will define high standards of care for advanced airway management in pre-hospital systems. The guidelines will focus on safe and practical delivery of PHEA, selection of the correct patient group, and governance standards. Indications for PHEA strongly recommended by the EHAC MWG are:

- ▪ Impending or actual hypoxia
- ▪ Impending or actual acute hypercapnia
- ▪ Threatened or actual loss of airway control
- ▪ Severe agitation associated with head injury
- ▪ Reduced level of consciousness

The EHAC MWG suggests that the requirement for, and provision of PHEA should be assessed on an individual case basis. Where PHEA is indicated, it should be performed in a timely fashion and should not significantly delay transfer of the patient to hospital. These guidelines are designed for physicians; paramedics performing pre-hospital drug-assisted intubation should meet the requirements of their employers and professional governing bodies.

## Training

**Standards for best practice:**

Only providers with competence and experience in the delivery of in-hospital drug assisted intubation should deliver PHEA.

- ▪ Physicians entering pre-hospital practice should have a minimum of 12 months experience of in-hospital anaesthetic practice and a minimum of 12 months experience in emergency medicine and acute medicine, before undertaking PHEA.
- ▪ Pre-hospital emergency care services should have a written standard operating procedure (SOP) for the conduct of PHEA. All relevant personnel must be fully conversant with this document.
- ▪ Pre-hospital emergency care services should provide appropriate training and competency assessment for all providers on an annual basis.
- ▪ All providers of PHEA should be competent in paediatric advanced airway management.
- ▪ The provider performing advanced airway management should be assisted by another member of the HEMS team with appropriate training for the safe delivery of PHEA, at all times.
- ▪ PHEA should be withheld if the HEMS team do not have the correct skillmix required to ensure safe and effective delivery of the procedure.
- ▪ Consultants in pre-hospital emergency medicine should be available for telephone advice at all times.
- ▪ All practitioners delivering PHEA should maintain a logbook of individual cases.

- ◦ **Further considerations:**
  - ▪ A relevant PHEA course or thorough induction training should be undertaken prior to starting clinical practice.

### EHAC MWG statement

It is well recognised that poorly performed tracheal intubation is associated with an increased morbidity and mortality [8, 9]. Repeated attempts at laryngoscopy increase the number of complications including severe hypoxia, regurgitation and aspiration, bradycardia and cardiac arrest [10, 11]. The minimum training requirement for pre-hospital practitioners delivering drug-assisted intubation remains controversial. Individual pre-hospital emergency care services should have pre-determined criteria for the minimum training requirements and competency levels before allowing personnel to practice PHEA. There is significant variation in intubation success rates for different groups of pre-hospital care providers with different skill mix. This was confirmed in a recent meta-analysis demonstrating a median intubation success rate for physicians of 99.1% compared with 84.9% for non-physician providers. In the non-physician group, PHEA significantly increased intubation success rate [12]. Prehospital tracheal intubation performed by the correct personnel, including experienced physician anaesthetists, nurse anaesthetists, or other appropriately trained healthcare providers, is associated with high success rates and a low incidence of complications [13]. One study suggested that a minimum of 57 intubations should be performed before providers are able to reliably perform successful tracheal intubation on a consistent basis. This study showed the intubation learning curve reached 90% success rate after a mean of 57 intubations but 18% of participants still required assistance after 80 intubations [14]. Available evidence for annual competency suggests there is a significantly higher incidence of difficult intubation amongst ‘proficient’ intubators who performed a median of 18 intubations per year, compared with ‘expert’ intubators who performed a median of 304 intubations per year (*p* < 0.05) [15]. EHAC MWG recommends that all practitioners in pre-hospital care should achieve a minimum of 80 supervised intubations, or spend the equivalent of one-year training in anaesthesia in a hospital setting before attempting pre-hospital drug assisted intubation. Maintaining currency in a pre-hospital environment can be challenging for any procedure [16]. All pre-hospital practitioners should routinely perform tracheal intubation within their regular professional capacity to ensure maintenance of essential skills.

## Planning

**Standards for best practice:**

- ▪ Environmental factors such as ambient light, noise and adverse weather conditions should be considered when deciding where and when to intubate the patient.
- ▪ Factors that may influence intubation success should be optimised prior to the first intubation attempt. These include good access to the patient (360-degree access where possible), and optimal positioning of the patient on an ambulance trolley placed at the correct height for the operator.
- ▪ Intubation should be performed prior to loading onto the aircraft unless adverse weather conditions prevent safe conduct. Intubation should only be performed in the aircraft provided there is no increased risk of an adverse event during the intubation procedure.
- ▪ Intubation must not be planned for, or performed in, the flight phase of aeromedical transfers.
- ▪ The triage decision and distance to destination hospital should be considered prior to intubation and discussed with the HEMS team.

- **Further considerations:**
  - ▪ Aviation regulations must be observed at all times.

### EHAC MWG statement

All factors influencing the success of an intubation attempt should be optimised prior to the first attempt including access to the patient, assembly of all required equipment, full monitoring and a verbalised management plan and triage decision. Whilst movement of sick or injured patients should be limited, the EHAC MWG strongly recommends that the patient should be moved to an area with adequate space to permit 360-degree access prior to intubation. Intubation should be performed outside the aircraft unless adverse events such as bad weather, low outside temperatures, or suboptimal light are considered likely to reduce the chances of a successful intubation. The patient should be placed on an ambulance trolley at an optimum height prior to any intubation attempts. The quality of laryngeal view, intubation and first pass success rates have been demonstrated to be optimal when the trolley is placed at chest height for the intubating clinician [17, 18]. Once the patient has been intubated they should be loaded onto the aircraft ensuring adequate access to the patient is maintained, with essential ‘rescue’ airway equipment easily available. The clinical condition of the patient should be reassessed each time the patient is moved to ensure the tracheal tube has not been misplaced or any deterioration has occurred requiring further intervention prior to take-off.

## Equipment

**Standards for best practice:**

The following equipment is considered essential for all intubation attempts and should be carried by personnel who are qualified to perform PHEA:

- ▪ Nasopharyngeal and oropharyngeal airways
- ▪ Two working laryngoscope handles with two different sized Macintosh blades
- ▪ Intubating bougie
- ▪ Cuffed tracheal tubes in appropriate sizes
- ▪ Spare tracheal tube (one size smaller)
- ▪ 10 or 20 ml syringe for cuff inflation (cuff checked prior to intubation)
- ▪ Tube tie or tube holder
- ▪ Bag-valve-mask with oxygen reservoir connected to oxygen
- ▪ Carbon dioxide monitoring (colorimetric and / or quantitative)
- ▪ Spare oxygen cylinder
- ▪ Suction
- ▪ Second generation supraglottic airway device (for failed intubation)
- ▪ Surgical airway equipment (e.g. scalpel / tracheal dilators / 6.0 tracheal tube / tube tie)
- ▪ Paediatric laryngoscopes with appropriately sized laryngoscope blades (size 1 and 2 Macintosh blades, and size 0 and 1 Miller blades are recommended)
- ▪ Uncuffed and cuffed tracheal tubes in appropriate size range for paediatric intubation

- **Further considerations:**
  - ▪ Videolaryngoscopy

### EHAC MWG statement

All pre-hospital systems must have all the required equipment available for each intubation attempt. The service should carry a range of laryngoscope blades and tracheal tubes in different sizes, appropriate for both adult and paediatric intubation. The tube size should be calculated prior to intubation. The use of a challenge-response equipment checklist is recommended.

Videolaryngoscopy is used in an attempt to improve laryngoscopic view and increase the overall intubation success rate and first pass intubation rate. The purpose of videolaryngoscopy is to enable all medical personnel involved to observe visualisation of the glottis and participate in improving the view where possible with interventions such as external laryngeal manipulation and suction. Data on the performance of these devices in improving intubation success rates are limited in this role. Most studies suggest videolaryngoscopes offer a benefit in pre-hospital intubation [19, 20], though certain conditions, such as blood in the airway, may impair performance [21]. Use of an intubating bougie is advocated in the management of difficult airways, usually if the view at laryngoscopy is poor or after one failed attempt at normal laryngoscopy [22, 23]. Routine use of a bougie for every intubation can also be considered good practice as it may optimize the first attempt at intubation.

## Conduct of the RSI

**Standards for best practice required:**

- ▪ PHEA should be performed using methods described in the standard operating procedure (SOP) for each individual service. Compliance with, or reasons for deviation from, the SOP should be formally documented.
- ▪ A formal checklist for PHEA should be carried out and include confirmation of monitoring, equipment, drugs, and failed intubation management.
- ▪ All required equipment should be assembled and checked prior to intubation.
- ▪ Drug doses should be calculated prior to intubation and confirmed with the anaesthetic assistant.
- ▪ Preoxygenation should be performed for at least 3 min before laryngoscopy.
- ▪ Each service should have, and be familiar with, a robust failed intubation plan.

- **Further considerations:**
  - ▪ Apnoeic oxygenation.

### EHAC MWG statement

There is increasing evidence for the benefit of both checklists and SOPs for many complex interventions. The introduction of such documents into pre-hospital practice has been shown to be feasible [24], and result in positive behavioural change to achieve the desired standardised care [25]. It is likely to be associated with a reduced rate of failed intubation [5, 26].

PHEA drugs should include an induction agent, an opioid and a rapid-onset muscle relaxant Careful consideration should be given to the type and dose of PHEA drugs, especially in unstable patients. Induction agents can also be omitted in imminent risk of cardiac arrest. Where possible the choice of agents used within individual services should be limited to improve familiarity with the PHEA process, promote the use of reproducible techniques, and reduce human error.

Passive apnoeic oxygenation with high-flow (15 l/min) oxygen via nasal prongs (or alternative methods) is a low-risk procedure. It is currently practiced by a number of pre- and in-hospital services around the world with demonstrable benefit in sustaining SaO_2_ above 95% during the apnoeic phase [27] and difficult laryngoscopy [28]. Nasal prongs should be applied to the patient in the preoxygenation phase in conjunction with a standard preoxygenation technique, and remain in situ during the period of drug-induced apnoea immediately prior to intubation. One pre-hospital service demonstrated a 6% reduction in patient desaturation rates during RSI following the implementation of apnoeic oxygenation [29]. Most studies demonstrate a benefit with apnoeic oxygenation, though some do not reflect this finding. A recent randomised controlled trial using 15 L/min of 100% oxygen via nasal cannulae compared to standard face mask preoxygenation failed to show any benefit [30] and the use of apnoeic oxygenation remains controversial.

The evidence for cricoid pressure is weak and relatively scarce. The authors recommend consideration of the use of cricoid pressure for PHEA under normal circumstances but practitioners should have a low threshold for removing it if the view at laryngoscopy is impaired.

External laryngeal pressure and manipulation may be of benefit when attempting to improve the view.

## Post RSI care and ventilation

**Standards for best practice:**

- ▪ Effective ventilation should be established and confirmed immediately following placement of the tracheal tube. Where available, a mechanical ventilator should be used in preference to hand ventilation, especially for longer transfers.
- ▪ The presence of an end-tidal carbon dioxide trace should be confirmed immediately after tube placement.
- ▪ The rate of ventilation should be titrated to end-tidal carbon dioxide.
- ▪ The position of tracheal tube should be confirmed and documented.
- ▪ The patient should be reassessed after each intervention or change of position, and before loading onto aircraft.
- ▪ The HEMS crew member(s) should have access to the patient during flight
- ▪ Intubation equipment and airway rescue equipment should be immediately available during flight.
- ▪ Normoxia and normocapnia should be achieved for each patient. Low to normocapnia should be considered for patients with traumatic brain injury (4.0–4.5 kPa; 30–35 mmHg).
- ▪ Ensure the patient is appropriately packaged with consideration given to clot stabilisation if bleeding, fracture immobilisation and maintenance of normothermia.
- ▪ Anaesthesia should be adequately maintained.
- ▪ The requirement for chest decompression should be considered.

- **Further considerations:**
  - ▪ Use of arterial blood gas monitoring.

### EHAC MWG statement

End-tidal carbon dioxide monitoring is mandatory for all intubated patients and lack of continuous capnography is considered to be associated with an increase in morbidity and mortality [9]. Inadequate (hypo- or hyper-) ventilation can be harmful in intubated trauma patients, and is believed to contribute to an increase in mortality in this patient group, particularly in patients with traumatic brain injury [31, 32]. Although outcomes for intubated ventilated trauma patients is undoubtedly multifactorial, good control of ventilation with prevention of hypoxia and hypo- or hypercapnia is likely to be of significant benefit.

Hypothermia in sick and injured patients is widely considered to be detrimental, contributing to systemic dysfunction. One study conducted in a pre-hospital setting observed higher rates of hypothermia (< 35 °C) in patients undergoing PHEA; the mortality rate was significantly higher in this patient group [33]. The presence of hypothermia in the pre-hospital setting is usually multifactorial and related to environmental exposure for the purposes of assessment and treatment, reduced metabolic function in skeletal muscle, and administration of intravenous fluids. Clinically significant effects on plasma coagulation and platelet function are seen at temperatures below 34 °C and the mortality from traumatic haemorrhage is markedly increased when core temperatures fall below 32 °C [34].

## Monitoring

**Standards for best practice:**

The following should be considered mandatory for all patients:

- ▪ Pulse oximetry.
- ▪ Noninvasive blood pressure.
- ▪ Heart rate.
- ▪ Continuous waveform and quantitative capnography.
- ▪ Continuous temperature monitoring.

**Further considerations:**

- ▪ Lactate

### EHAC MWG statement

Full monitoring should be attached to the patient prior to induction of anaesthesia and a summary of the information obtained from the monitoring recorded in the patient’s documentation [35]. The temperature of the patient should be closely monitored to avoid unwanted hypothermia which may exacerbate acute traumatic coagulopathy [36], or for targeted measurement for patients in whom therapeutic hypothermia is considered to be of benefit.

## Special circumstances

- ▪ Night operations
- ▪ Adverse weather or environmental conditions
- ▪ Psychiatric patients
- ▪ Pregnant patients
- ▪ Children

Pre-hospital management of sick and injured patients is associated with a wide variety of challenges and factors that may influence individual patient management. It would be impossible to produce guidelines that accounted for all variables that may be encountered. Certain circumstances may require deviation from best practice guidelines. All decisions about how to proceed should be case specific and following a dynamic risk-benefit assessment and with a senior clinician providing support for the decision-making process.

## Key performance indicators

The practice of PHEA is increasing and adequate data collection is essential to improve practice through local audit and clinical governance processes. The following variables have been suggested as part of the minimum dataset for collection and analysis [37].

**System variables:**

- Highest level of EMS provider on scene
- Airway equipment available
- Anaesthetic agents available
- Method of transportation
- Response time
- Provision of adequate governance structure

**Patient variables:**

- Age
- Gender
- Co morbidity
- Patient category
- Indication for airway intervention
- Vital signs pre induction of anaesthesia (Heart rate, respiratory rate, GCS, systolic blood pressure, oxygen saturation)

**Post intervention variables:**

- Post intervention ventilation
- Vital signs post induction of anaesthesia (End tidal carbon dioxide, heart rate, systolic blood pressure, oxygen saturation)
- Survival status
- Number of attempts at airway intervention
- Complications (hypoxia, hypotension, arrhythmias / bradycardia, aspiration, misplaced tracheal tube, oesophageal intubation (recognised / unrecognised), cardiac arrest)
- Drugs used to facilitate procedure
- Overall intubation success rate
- Devices used in successful airway management

**Further considerations:**

- Intubation success rate at first attempt
- Management of failed intubation

All patient documentation should be completed and data collection should be tailored to the requirements and processes of individual systems.

### Summary statement

EHAC MWG recommends a standardised approach to pre-hospital emergency anaesthesia and advanced airway management described in a clear and simple Standard Operating Procedure that is followed by competent clinicians. Only personnel with sufficient experience and expertise should deliver pre-hospital advanced airway management. Standards for the pre-hospital procedure should not be inferior to those found in-hospital regarding equipment availability, patient monitoring and post-intubation care. Continuous audit of the Key Performance Indicators is essential to maintain these standards.
